# The Book World of Medicine and Science

**Published:** 1903-06-13

**Authors:** 


### THE BOOK WORLD OF MEDICINE AND SCIENCE.

GENITO-URINARY SURGERY AND VENEREAL DISEASES. By THO. WHITE and E. MARTIN. Fifth Edition. Pp. 1,068. 251 illustrations. (Philadelphia and London: The Lippincott Company. 1902. 21s. net.)

THIS magnificent text-book, the joint production of Drs. White and Martin, the distinguished Pennsylvanian surgeons, is in every way a model of what such a book should be. Its external appearance is handsome, the printing and paper leave little or nothing to be desired, and the illustrations, as artistic as the subject will allow, really do illustrate. The subject-matter of the book embraces a study of the injuries and diseases of the male genital organs, a complete account of venereal diseases, a description and discussion of the injuries and diseases of bladder and kidneys, and a sufficient elucidation of the purely sexual disorders and abnormalities. The book is well written, the language well chosen, and—without offence, be it said—as free from the presence of objectionable Americanisms as it is from Teutonic pedantry. It is an advantage, we think, that the very adequate anatomical descriptions preface particular chapters and not the whole work, and that the pathological disquisitions are not burdened by references to the mistaken notions of the earlier searchers after truth, but are simple, straightforward expositions of those views which find favour with most well-balanced minds. The practitioner and student alike will appreciate the definiteness with which plans of treatment are stated, and the generosity with which prescriptions are set forth. These features of Drs. White and Martin's book are particularly welcome to those who turn for information to so many current text-books, only to find bald categories of drugs and alternative methods. As an excellent example of the authors' common sense and logical attitude we would refer to the chapter on syphilitic heredity. In this chapter the actual facts, so far as we know them, are put forward, with some novelty of expression it is true, but divorced from the obscurity and vague speculation of so many other authors. Drs. White and Martin have certainly not overburdened their work with references to the labours of others, but at the same time they have been careful not to omit acknowledgment where it is plainly due, and every opinion almost that it is of importance to be acquainted with is recorded in their pages. The attitude of the authors to the difficult questions of ethics and morality necessarily brought forward in such a book as this is, we think, unexceptionable, and they have not shrunk from recognising the insoluble nature of the problems raised when nature and society are in conflict. The unpleasant but unavoidable subject of sexual perversion and abnormality is treated at sufficient length, but with perfect dignity and reticence. It may be said, perhaps, that our account of this book is too eulogistic to be of value as a criticism. Yet we are confident that our opinion will not, by those who read the book, be dissented from, and that all those who do once read the book will place it in a position on their bookshelves, convenient for the frequent reference they will make to it.

OPERATIVE AND CLINICAL SURGERY. By the late Sir WILLIAM STOKES, M.D., M.Ch., F.R.C.S.I. Edited by WILLIAM TAYLOR, M.B., F.R.C.S.I. (London: Baillière, Tindall and Cox. 1902. Pp. 484. Illustrations 52, and 26 plates. 10s. net.)

THERE is something always pathetic in the publication of the monographs of a well-known surgeon who has departed this life. Those of the readers of this volume who had the pleasure of knowing the late Sir William Stokes will echo the words of Professor Ogston, the writer of the memoir which introduces the collection of articles contained in this book. He says "He lived surrounded by scenes to which his heart went out, by friends more loving than most can attract, and he died for his country and his country's cause." Thirty-six chapters follow, embodying a selection of his writings which it would have been a loss to surgery not to have recorded. The range of subjects with which they deal proves the wide view and knowledge possessed by their author. Many of these were thoroughly advanced, and in fact ahead of the times, when they were penned by him, and show such a grasp of the matters that they deal with that one can recall the strong personality of the writer. To mention but a few, aneurism, amputation at the hip-joint, pylorectomy, rupture of the female bladder, and astragaloid osteotomy in the treatment of flat-foot, is sufficient to show that the work teems with interest. In one of the chapters, Sir William gives some weighty views with regard to surgery of the thorax. Speaking of Esthlander's operation, he says: "From what I have experienced in my own practice, and seen in that of others, the conviction has been forced upon me that we are only, so to say, commencing to tread what is as yet but a dimly lit, and to a great extent unexplored path, one, however, destined to lead, notwithstanding many probable failures and disasters in the future, to far greater achievements than have hitherto been accomplished by even the most conscientious and skilful surgeons.' Such a prophecy is surely and steadily seeing its fulfilment, and its propounder was not one of the least to bring about its consummation. To all who look for sound common sense, practical details based upon carefully thought over personal experience, we should recommend the perusal of this volume. It is closed by some papers written in South Africa shortly before his lamented death.

THE POPULAR ILLUSTRATED GUIDE TO THE SOUTH-EASTERN AND CHATHAM RAILWAY COAST RESORTS. By W. T. PERKINS. (London: McCorquodale and Co., Ltd. 6d.)

THIS volume is well printed, on good paper, and contains many admirable illustrations of the watering-places served, both in England and in France, by the South-Eastern and Chatham Railway. It is, moreover, exceedingly interesting in itself, and contains a great deal of historical and antiquarian lore. Even one who has not visited the south-eastern parts of England—on whose shores first Cæsar and afterwards St. Augustine made their landing when in war and in peace respectively they came to conquer our forefathers —will find this guide-book more than worth the price asked for it, and those who have visited any of the resorts mentioned, without first reading it, will soon discover that they have missed so much for lack of it that they must go back again. As an advertisement of the railway it should be invaluable, and as a contribution to the history of a part of England that is full of interest it is worth possessing by the stay-at-home.

EVERYBODY'S STREET GUIDE TO LONDON. (John Dicks, Effingham House, Arundel Street, Strand, W.C. 1d.)

THIS is such a useful little book that one wonders that no one thought of it before. In parallel columns are given the names of every street in London, that of the nearest main thoroughfare, and that of the nearest railway station. People who have suffered—and who has not?—from pressing invitations to go to some street or terrace with no better guide as to its direction than the letters which indicate its postal district, will welcome this handy pennyworth, and we should think that before long "Everybody's Street Guide to London," will be in everybody's hands.

